# Comparison of computational fluid dynamics with transcranial Doppler ultrasound in response to physiological stimuli

**DOI:** 10.1007/s10237-023-01772-9

**Published:** 2023-10-08

**Authors:** Harrison T. Caddy, Hannah J. Thomas, Lachlan J. Kelsey, Kurt J. Smith, Barry J. Doyle, Daniel J. Green

**Affiliations:** 1 Vascular Engineering Laboratory, Harry Perkins Institute of Medical Research, Queen Elizabeth II Medical Centre, Nedlands, Australia and the UWA Centre for Medical Research, The University of Western Australia, Perth, Australia; 2 School of Human Sciences (Exercise and Sport Sciences), The University of Western Australia, Perth, Australia; 3 School of Engineering, The University of Western Australia, Perth, Australia; 4 Cerebrovascular Health, Exercise, and Environmental Research Sciences Laboratory, University of Victoria, Victoria, Canada

**Keywords:** Cerebral vasculature, Computational fluid dynamics, Transcranial Doppler ultrasound, Stimuli

## Abstract

Cerebrovascular haemodynamics are sensitive to multiple physiological stimuli that require synergistic response to maintain adequate perfusion. Understanding haemodynamic changes within cerebral arteries is important to inform how the brain regulates perfusion; however, methods for direct measurement of cerebral haemodynamics in these environments are challenging. The aim of this study was to assess velocity waveform metrics obtained using transcranial Doppler (TCD) with flow-conserving subject-specific three-dimensional (3D) simulations using computational fluid dynamics (CFD). Twelve healthy participants underwent head and neck imaging with 3 T magnetic resonance angiography. Velocity waveforms in the middle cerebral artery were measured with TCD ultrasound, while diameter and velocity were measured using duplex ultrasound in the internal carotid and vertebral arteries to calculate incoming cerebral flow at rest, during hypercapnia and exercise. CFD simulations were developed for each condition, with velocity waveform metrics extracted in the same insonation region as TCD. Exposure to stimuli induced significant changes in cardiorespiratory measures across all participants. Measured absolute TCD velocities were significantly higher than those calculated from CFD (*P* range < 0.001–0.004), and these data were not correlated across conditions (*r* range 0.030–0.377, *P* range 0.227–0.925). However, relative changes in systolic and time-averaged velocity from resting levels exhibited significant positive correlations when the distinct techniques were compared (*r* range 0.577–0.770, *P* range 0.003–0.049). Our data indicate that while absolute measures of cerebral velocity differ between TCD and 3D CFD simulation, physiological changes from resting levels in systolic and time-averaged velocity are significantly correlated between techniques.

**Supplementary Information:**

The online version contains supplementary material available at 10.1007/s10237-023-01772-9.

## Introduction

To maintain adequate oxygenation, nutrient supply and waste disposal, the cerebral vasculature is homeostatically regulated in response to physiological stressors such as hypercapnia and exercise (Ozturk and Tan 2018; Querido and Sheel 2007; Smith and Ainslie 2017). Assessment of cerebrovascular health is typically undertaken via measurement of changes in the function of intra-cranial arteries such as the middle cerebral artery (MCA) (Bae et al. 2014; Birch et al. 1995; Meng et al. 2019). Assessment of MCA velocity and localised haemodynamics are therefore important for diagnosis of cerebrovascular health, as well as physiological research.

One established method for directly measuring blood velocity, diameter and flow within the brain is magnetic resonance imaging (MRI)—where protocols such as magnetic resonance angiography (MRA) or 4D flow MRI are applied. MRI scans possess relatively high spatial resolution and are able to capture the complex 3D structure and flow patterns within cerebral arteries (Ainslie and Hoiland 2014). However, during conventional MRI scanning individuals are generally required to be in a supine or prone position for relatively long periods (10–20 min) (Markl et al. 2012; Stankovic et al. 2014; Zarrinkoob et al. 2021), which limits the technique when external stimuli such as hypercapnia or exercise are of interest.

In contrast, computational fluid dynamics (CFD) simulations can provide an alternative method for determining the 3D haemodynamics within cerebral arteries. Although not directly measuring flow, CFD simulations calculate the momentum and pressure within a fluid domain and can be useful in situations where direct assessment is not possible. Consequently, CFD simulations represent an attractive approach for characterisation of rapid changes in cerebrovascular haemodynamics (e.g. in response to physiological stimuli), as they can leverage the high spatial resolution associated with 3D MRA imaging alongside the high temporal resolution of duplex ultrasound, that furthermore can be measured in the internal carotid (ICA) and vertebral (VA) arteries to provide inlet flow conditions (Thomas et al. 2020). In addition to estimating cerebral artery velocities, CFD simulations can also provide useful velocity-derived haemodynamic information, such as localised 3D wall shear stress distributions and indications of oscillatory shear, enabling further understanding of the interactions of blood flow with the surrounding vessel endothelium.

Transcranial Doppler (TCD) is an ultrasound technique which is commonly used for measuring intra-cranial blood flow velocity (BFv). TCD is non-invasive, is reproducible and can be used in real time with high temporal resolution (Willie et al. 2011). Due to its portability and low cost, it is used extensively to better characterise cerebrovascular responses to physiological stimuli including hypercapnia, hypocapnia, exercise, vessel occlusion, shear-mediated endothelial responses and neurovascular coupling (Bishop et al. 1986; Coverdale et al. 2014; Hoiland et al. 2017; Phillips et al. 2016; Thomas et al. 2020). Despite these benefits, TCD can only measure blood flow velocity due to resolution constraints which prevent B-mode imaging of arterial diameter (Willie et al. 2011). Additionally, TCD measures are angle dependent. Furthermore, many devices use standard angle correction factors independent of individual vessel geometries which could influence the angle of insonation. Determination of blood flow, and therefore the localised 3D haemodynamics, remains a limitation when using TCD. While velocity in the MCA can be measured directly via TCD or calculated indirectly via flow-conserving 3D CFD simulations, and although comparisons between these approaches have been undertaken under static conditions (Groen et al. 2018; Panerai et al. 2016; Rivera et al. 2016; Shen et al. 2020), few studies have compared CFD and TCD methods in response to physiological stimuli (Coverdale et al. 2014).

In this study, we compared velocity waveform responses to the stimuli of hypercapnia and submaximal exercise between simulated cerebrovascular velocities calculated using CFD methods and independently measured TCD ultrasound. Our CFD approach combined ICA and VA duplex ultrasound measurements to prescribe volumetric flow inlet boundary conditions and used 3D geometries based on participant-specific MRA cerebrovascular reconstructions. We hypothesised that flow-conserving CFD and TCD-derived waveform velocity metrics would be similar in absolute terms and correlated at rest and in response to physiological stimuli.

## Materials and methods

### Participant cohort and medical imaging

The experimental procedures used in this study were approved by the University of Western Australia Human Research Ethics Committee. A total of 12 healthy participants (six females and six males) were recruited for this study with ages ranging between 19 and 28 years. Participants were made aware of the experimental procedure and associated risk. Written consent was obtained for each participant prior to commencement of the experimental study. Prior to cerebrovascular stimuli, each participant underwent a 3 T time-of-flight MRA (Siemens MAGNETOM Skyra) neck and head scan. This scan had a pixel size of 0.31 mm and a slice thickness of 0.75 mm.

### Cerebrovascular stimuli procedure

Participants were exposed to conditions previously described by Thomas et al. (2020), consisting of rest (5-min recumbent), hypercapnia (5 min of 6% CO_2_ via Douglas bag recumbent) and submaximal exercise (5-min recumbent cycling at 90 Watts) conditions, respectively. Each session was conducted in the morning for all participants, who were instructed to fast (no food, tea or coffee) and abstain from consumption of alcohol or performing exercise in the 24 h prior to the session. At the end of each of the conditions, we simultaneously measured velocity (Doppler ultrasound) and intraluminal diameter (B-mode ultrasound) in the left and right ICAs and VAs using two identical 10-MHz linear array probes and high-resolution ultrasound machines (Terason 3200, Teratech, Burlington, MA) using standardised search techniques (Willie et al. 2011). Continuously during each condition, we measured the velocity envelope in the right M1 segment of the MCA via the middle transtemporal window using a 2-MHz TCD probe (TCD, Spencer Technologies, Seattle, WA) which was held in place with a headpiece (M600 bilateral head frame, Spencer Technologies) as per methods described previously (Hoiland et al. 2017).

Throughout all exposure conditions, we continuously measured end-tidal partial pressure of CO_2_ (PETCO_2_) and O_2_ using a gas analyser (Gas Analyzer, ADInstruments, New South Wales, Australia). For analysis and calculation of ICA and VA flows and TCD MCA velocities, we considered steady-state data sampled within the final 30-s period of each 5-min exposure condition. To mitigate cerebral priming, participants underwent a 10-min washout period. This involved participants remaining in a recumbent position between each of the exposure conditions, which was sufficient to return metrics (arterial pressure, heart rate, PETCO_2_, time-averaged MCA velocity from TCD) to baseline levels across all participants.

### MRA reconstruction and ultrasound analysis

#### MRA 3D reconstruction

To create the 3D fluid domain for the CFD simulations, we imported the DICOM images from the MRA scans into in-house image reconstruction software. We used region-growing techniques to select similar intensity labelled pixels greater than an intensity value of 200 to outline the fluid contained within the cerebrovascular geometry. The software used an inbuilt marching cubes algorithm to create a 3D isosurface. This isosurface was globally smoothed to within 5% of the starting volume, which was then imported into STAR-CCM+ (v12, Siemens, Munich, Germany) to perform surface repair, remove reconstruction artefacts and perform local smoothing. Outlets were truncated perpendicular to the vessel centreline at least two bifurcations downstream from the Circle of Willis (CoW).

#### Duplex and TCD ultrasound

The duplex ultrasound measurements of diameter and velocity at the ICAs and VAs over three cardiac cycles (Kurmanavichius et al. 1989) were converted to time-varying volume flow rates assuming Poiseuille flow and waveform ensemble averaged and processed as described previously (Tallon et al. 2022; Thomas et al. 2020). This resulted in spline fitted and peak aligned ensemble averaged waveform data for the resting, hypercapnia and exercise conditions. Total cerebral blood flow (tCBF) was calculated as the sum of the time average of the flow waveforms in the ICAs and VAs. Similarly, for each participant envelope TCD velocity waveform data from three cardiac cycles within the right M1 segment were combined into an ensemble averaged waveform using the same process.

### Computational fluid dynamics

#### Computational mesh

The simulations were developed in the commercial CFD package STAR-CCM+ (V12, Siemens, Germany) and followed recommendations for development of cerebrovascular simulations (Berg et al. 2019). We used a combination of a polyhedral element mesh for the core of the fluid domain and 20 prism layer elements in the near wall boundary to sufficiently capture the velocity gradients and ensure accurate calculation of wall shear stress. In addition, we prescribed extrusions at the fluid boundaries equal to 11 times the boundary diameter to ensure adequate development of flow upstream and downstream of the fluid domain (Bluestein et al. 1997). Mesh core density was set proportional to local vessel diameter, and the mesh settings were prescribed as per our previously published work (Thomas et al. 2020). These settings were optimised to ensure mesh independence using the grid convergence index (Roache 1994) and used the subject with the greatest inlet velocity measurements for this optimisation. Optimal mesh size settings were deemed sufficient when the grid convergence index for wall shear stress within the CoW was found to fall below 3% (Thomas et al. 2020). Final mesh sizes ranged from 9.1 to 16.7 million cells per geometry. Further details regarding the computational mesh and extrusion specification are given in online resource 1.

#### Boundary conditions

Boundary condition specification followed methods as described previously (Thomas et al. 2020); however, they are described in greater detail in online resource 1. Briefly, the volumetric flow waveforms derived from measured duplex ultrasound at the ICAs and VAs were converted to mass flow waveforms assuming a fluid density of 1050 kg m^−3^ (Levitt et al. 2017). The measured mass flow waveforms for the rest, hypercapnia and submaximal exercise conditions were all prescribed at the corresponding extruded inlet boundary using a plug flow condition, which then allowed for the natural development of flow throughout said extrusions prior to entering the subject-specific cerebrovascular 3D geometry. Outlet boundary conditions were implemented using the WALNUT code described previously (Thomas et al. 2020), which initially estimates the splitting of blood flow exiting the fluid domain into seven regions (left and right posterior, left and right middle, anterior, cerebellum and ophthalmic arteries), with different flow distributions to these regions based on the average volumetric flow measured at the ICAs and VAs. Within these seven regions within the brain, flow was then split using an adaptation of the Murrayʼs law formulation (Chnafa et al. 2018) as defined in Eq. 1, using an exponent of *n* = 2.33 (Thomas et al. 2020).

1 $$Q_{{{\text{out}},i}} = Q_{{{\text{in}},{\text{region}}}} \times \frac{{d_{i}^{n} }}{{\sum\nolimits_{j = 1}^{N} {d_{j}^{n} } }}$$

where *Q*_out*,i*_ is the flow out of outlet *i*, *Q*_in,region_ is the WALNUT calculated flow into the region of the cerebral vasculature which is shared by outlet *i* as described previously (Thomas et al. 2020), *d* is the outlet diameter and *n* is the flow split exponent.

#### Physical assumptions

Blood was assumed to be incompressible with a density of 1050 kg m^−3^ (Levitt et al. 2017). The non-Newtonian nature of blood viscosity was modelled using the Carreau–Yasuda viscosity model using parameters appropriate for blood flow leading to and within the CoW (*η*_*∞*_ = 0.0022 Pa s; *η*_*0*_ = 0.022 Pa s; *λ* = 0.11 s; *a* = 0.644; *n* = 0.392) (Kim et al. 2006). We assumed the arterial walls were rigid along with a no-slip wall boundary condition and used a laminar flow regime in line with previous cerebrovascular simulations (Alnæs et al. 2007; Kim et al. 2006; Shojima et al. 2004; Thomas et al. 2020).

#### Simulation execution

Simulations ran for three consecutive cardiac cycles to ensure flow stabilisation, with results extracted from the fourth cardiac cycle. We used an implicit unsteady segregated flow solver and the Euler implicit backwards difference second-order temporal discretisation scheme with an automated time-step control which permitted the time-step to vary between 0.001 and 0.005 s depending on the Courant number. New time steps were triggered if absolute continuity and momentum residuals fell below a value of 10^−9^, or if the number of inner iterations reached 50. Our simulations were executed using the STAR-CCM+ finite-volume method on Magnus, a Cray XC40 supercomputer (Pawsey Supercomputing Centre, Perth, Australia) housing a total of 1488 compute nodes each containing 24 cores per node. We ran each simulation utilising 25 nodes over a collective of 500 cores. The rest, hypercapnia and exercise simulations required an average of 3000, 2800 and 2000 core hours, respectively, to run, which equated to total simulation run times ranging from approximately 3–7 h.

### Data collection, analysis and statistics

**Fig. 1:**
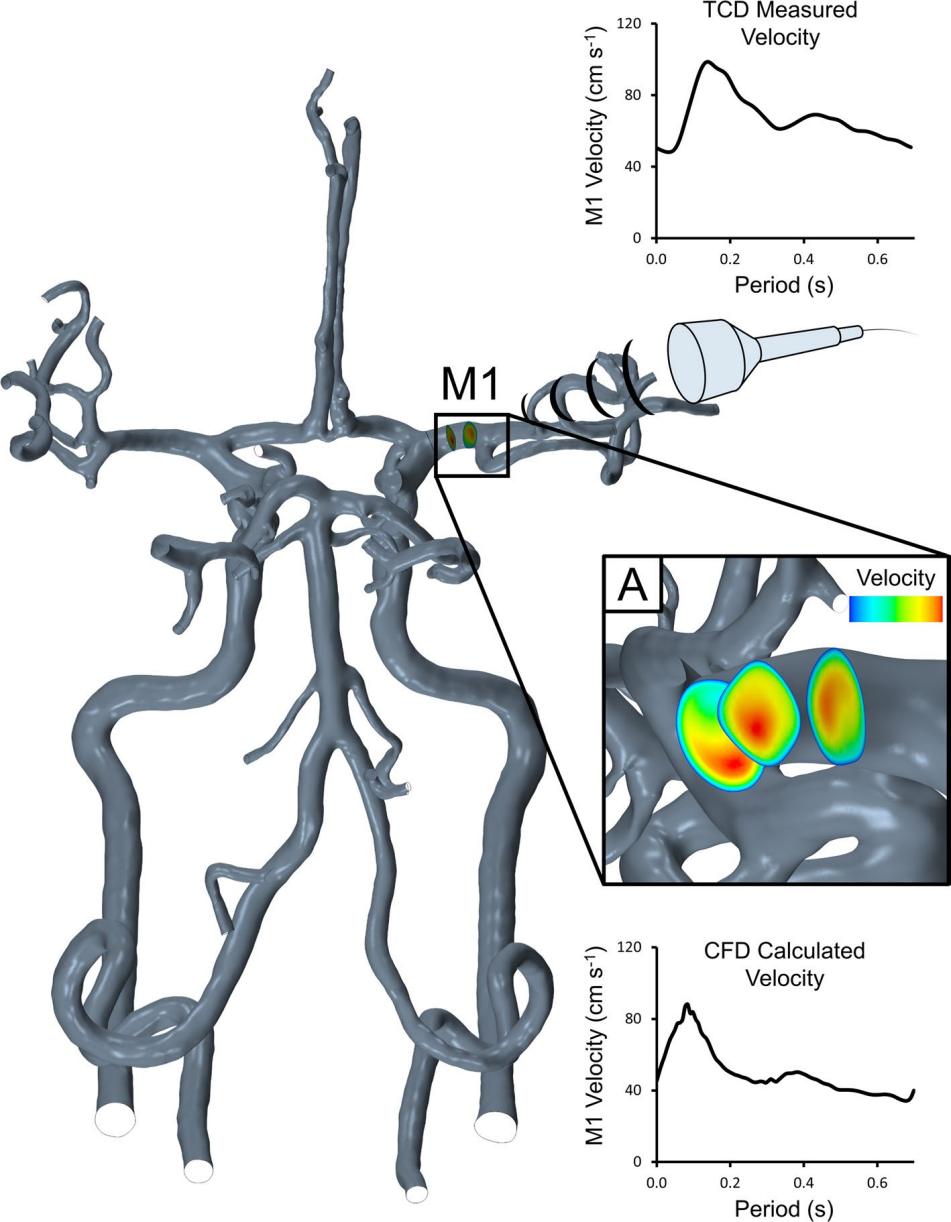
Example TCD probe as well as consecutively spaced CFD constrained planes **A** demonstrating sampling of data within the right M1 segment. Within the CFD simulations, maximal velocity was calculated and extracted at each of these planes and averaged into a mean value representative of the peak velocity envelope from TCD insonation of the right M1 segment in each participant. Example velocity waveforms from TCD and CFD sources from an individual are provided. Velocity waveform metrics were then extracted and compared between sources across a range of different exposure conditions (rest, hypercapnia and exercise)

Simulations were allowed to stabilise over three cardiac cycles, after which we extracted maximal BFv waveforms from each simulation over the fourth cardiac cycle using the average of the maximum value from three consecutively spaced constrained planes located within the right M1 segment (Fig. 1) to represent the region insonated using TCD. The velocity waveforms extracted from the simulation and from the TCD ultrasound envelope for each participant and exposure condition were analysed for their characteristics using a custom MATLAB script (R2016, MathWorks, Natick, MA). We extracted the systolic, cardiac cycle time-averaged and end-diastolic velocities from the envelope of these waveforms.

We used paired *t*-tests to test for significant differences between inter-source distributions as well as intra-source hypercapnia or exercise data compared to resting data. Pearsonʼs correlation and Bland–Altman plots were used to investigate the correlations between absolute and relative change CFD and TCD velocity waveform data for each participant in response to different stimuli. Normality was tested using the Shapiro–Wilk test. Where applicable, data are presented as group mean ± standard deviation. Statistical significance was assumed for *P*-values where *P* < 0.05.

## Results

### MRA reconstructions and participant cardiorespiratory responses

**Fig. 2:**
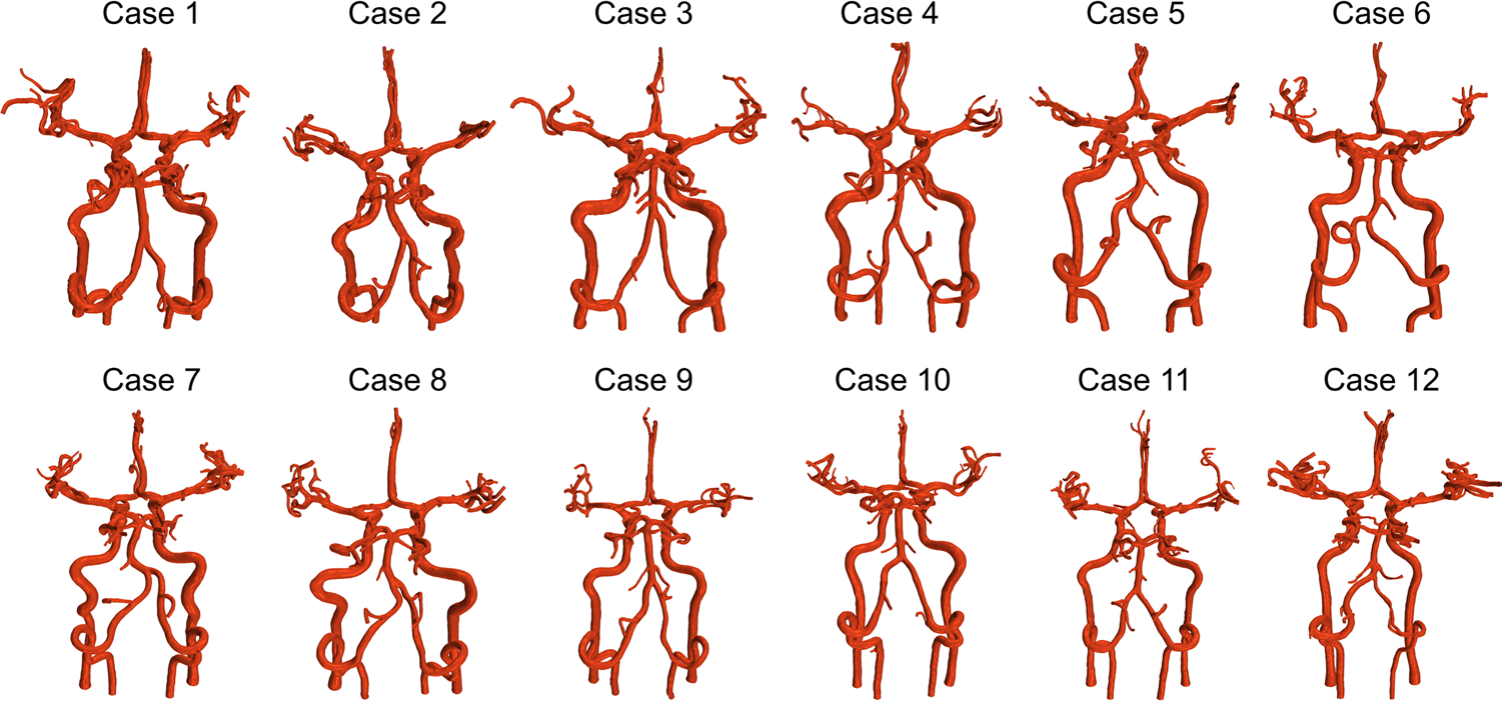
MRA-derived 3D cerebrovascular reconstructions of each participant

Participants had an average age, BMI and VO_2_max score of 22.9 ± 3.4 years, 21.6 ± 2.9 kg m^−2^ and 44.7 ± 9.4 mL kg^−1^ min^−1^, respectively. Reconstructed 3D models from MRA data of each of the 12 cases all exhibited a complete CoW and are presented in Fig. 2. Participant cardiorespiratory responses including PETCO_2_, mean arterial blood pressure (MAP), heart rate (HR) as well as ultrasound-derived time-averaged volumetric blood flow at the ICAs and VAs as summed tCBF under resting, hypercapnia and exercise conditions are displayed in Table 1. Compared to rest, hypercapnia significantly increased all cardiorespiratory measures and blood flows (*P* range < 0.001–0.008). Although exercise also significantly increased all cardiorespiratory measures (PETCO_2_, MAP and HR; *P* range < 0.001–0.042), blood flow to the brain did not significantly increase from rest (*P* range 0.051–0.868). Individual CFD and TCD velocity waveform data for resting, hypercapnia and exercise conditions for each case are given in online resource 2.

**Table 1:** Participant cardiorespiratory responses under different conditions

	Rest (mean ± SD)	Hypercapnia (mean ± SD)		Exercise (mean ± SD)
PETCO_2_ (mmHg)	42.2 ± 2.5	50.7 ± 1.9^††^		43.8 ± 4.2^†^
MAP (mmHg)	100.0 ± 8.1	106.0 ± 11.8^†^		124.8 ± 15.2^††^
HR (bpm)	68.7 ± 9.5	74.1 ± 8.1^†^		120.4 ± 21.3^††^
Blood flow (mL min^−1^)				
LICA	268.9 ± 63.3	374.6 ± 79.3^††^		272.8 ± 67.2
RICA	218.8 ± 74.2	308.6 ± 79.3^†^		262.9 ± 50.3
LVA	74.2 ± 26.4	119.4 ± 30.9^††^		79.7 ± 27.1
RVA	56.2 ± 18.3	97.0 ± 32.3^††^		68.4 ± 34.0
tCBF (mL min^−1^)	618.1 ± 129.7	899.7 ± 128.4^††^		683.7 ± 124.3

Data are mean ± standard deviation. PETCO_2_ = end-tidal partial pressure of CO_2_; MAP = mean arterial pressure; HR = heart rate; LICA = left internal carotid artery; RICA = right internal carotid artery; LVA = left vertebral artery; RVA = right vertebral artery; tCBF = total cerebral blood flow. Crosses (^†^) indicate the level of significance (^†^*P* < 0.05; ^††^*P* < 0.001) compared to rest

### Absolute and relative changes in total cerebral flow and MCA velocity

**Fig. 3:**
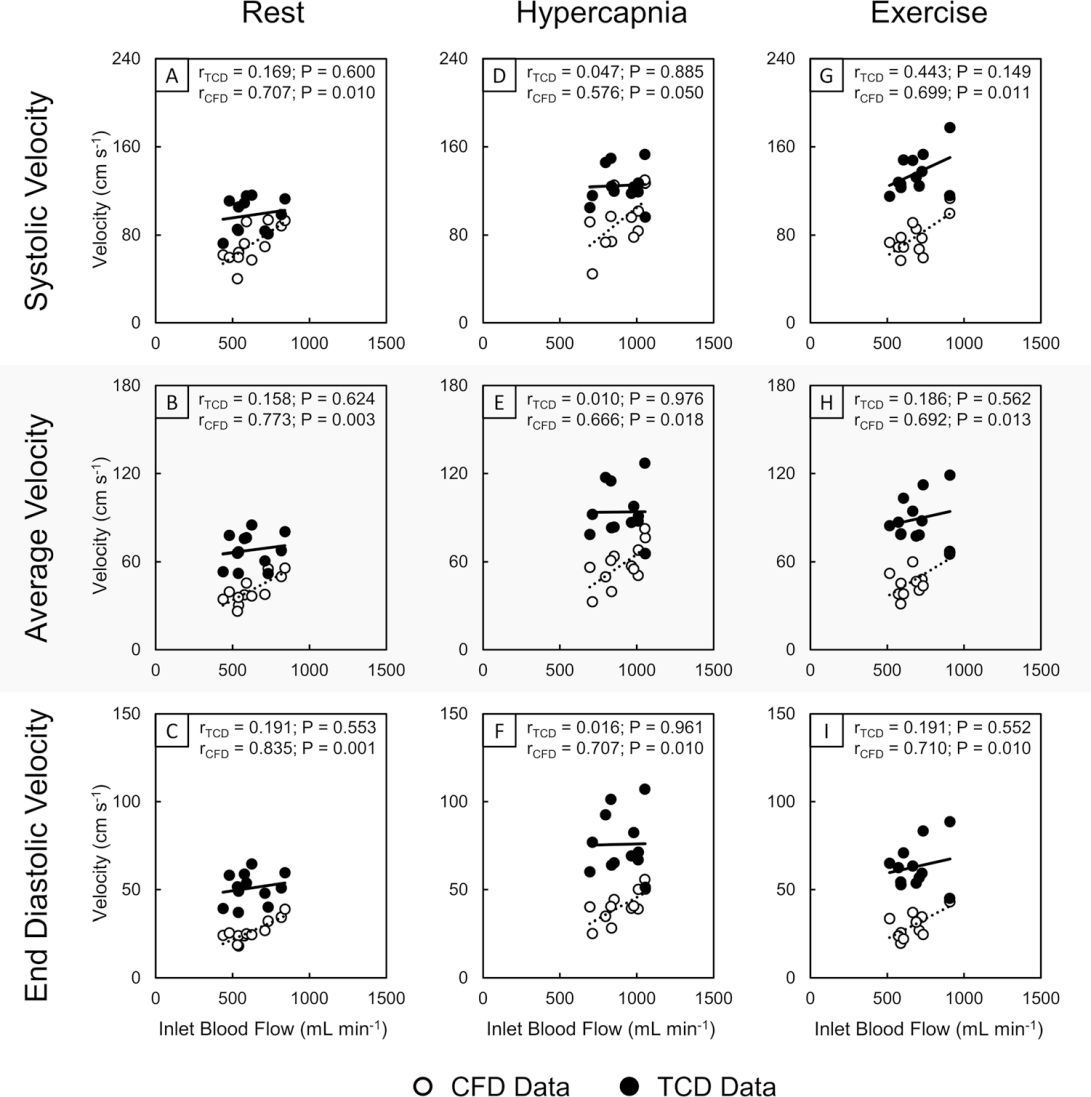
Correlation plots of inlet blood flow (tCBF) with the velocity waveform characteristics of systolic (**A**, **D**, **G**), average (**B**, **E**, **F**) and end-diastolic (**C**, **F**, **I**) maximal velocity extracted from CFD (white) and TCD (black) data in the right M1 segment of the MCA for the conditions of rest (**A–C**), hypercapnia (**D**–**F**) and exercise (**G**–**I**). Pearsonʼs correlation coefficient (*r*) and *P*-value (*P*) are displayed for each data source per correlation plot

Correlations between absolute velocity metrics from CFD and TCD sources with tCBF calculated from the ICA and VA flow data across all stimuli were compared (Fig. 3). Correlations of tCBF to velocity from the CFD data were all significantly and positively correlated (*r* range 0.576–0.835, *P* range 0.001–0.050), while TCD data yielded very mild positive correlations that were not significant (*r* range 0.010–0.443, *P* range 0.149–0.976), irrespective of exposure conditions or velocity metric.

**Fig. 4:**
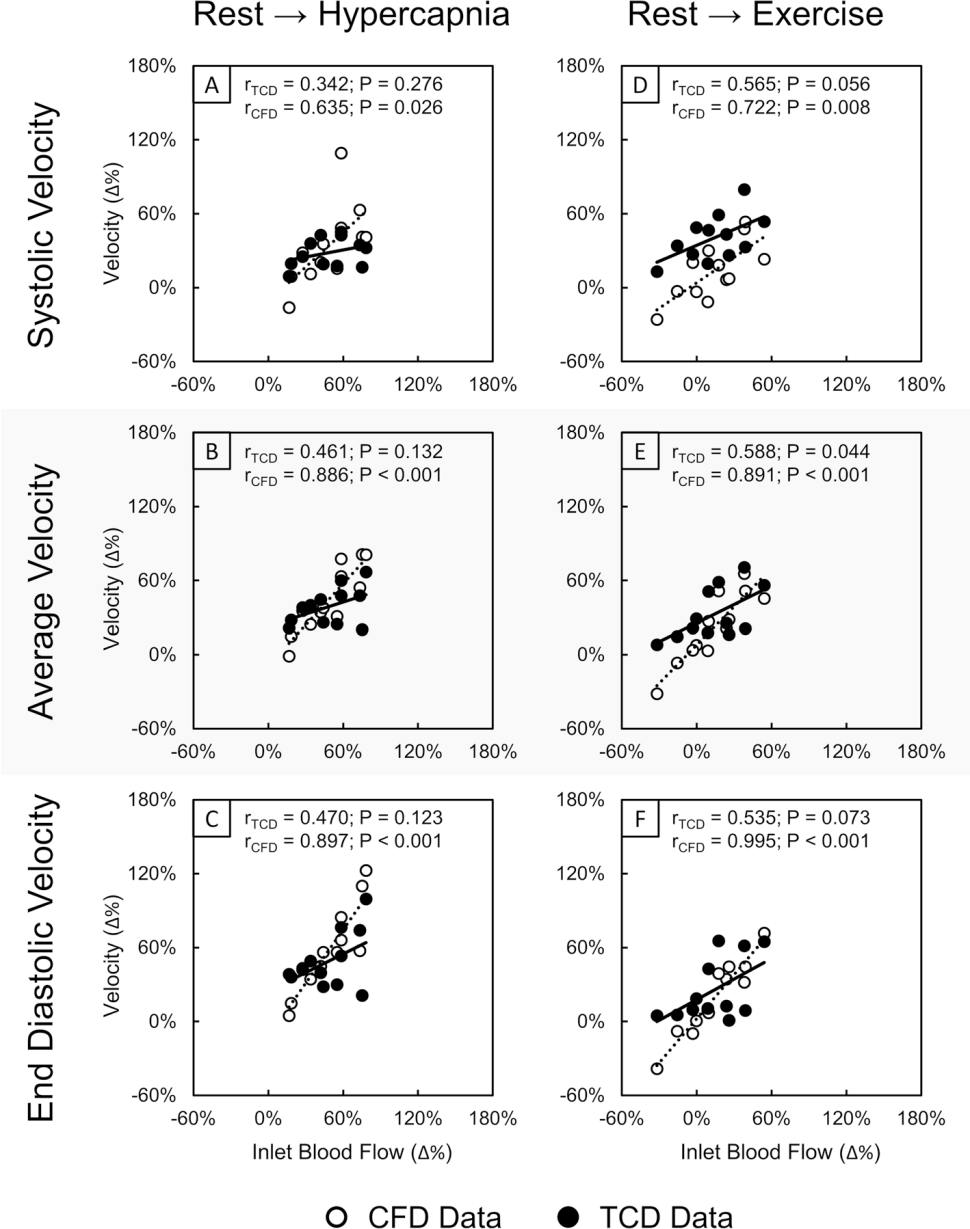
Correlation plots of the relative change (Δ%) in inlet blood flow (tCBF) with the relative change in systolic (**A**, **D**), average (**B**, **E**) and end-diastolic (**C**, **F**) maximal velocity extracted from CFD (white) and TCD (black) data in the right M1 segment of the MCA for responses from rest to hypercapnia (**A**–**C**) and rest to exercise (**D**–**F**). Pearsonʼs correlation coefficient (*r*) and *P*-value (*P*) are displayed for each data source per correlation plot

We also investigated correlations between relative changes in velocity metrics from CFD and TCD sources with the relative changes in tCBF in response to stimuli (Fig. 4). We again observed strong positive and significant correlations between the relative change in tCBF and MCA velocity calculated from CFD simulations across both responses to hypercapnia and exercise (*r* range 0.635–0.955, *P* range < 0.001–0.026). Despite correlations of relative changes in TCD velocity metrics with tCBF remaining positive and generally becoming stronger, outside of average velocity from rest to exercise (Fig. 4E: *r* = 0.588, *P* = 0.044), these correlations still remained mild and were not significant (*r* range 0.158–0.535, remainder *P* range 0.056–0.276).

### Absolute MCA velocity data distributions and correlations at rest and during physiological stimuli

**Fig. 5:**
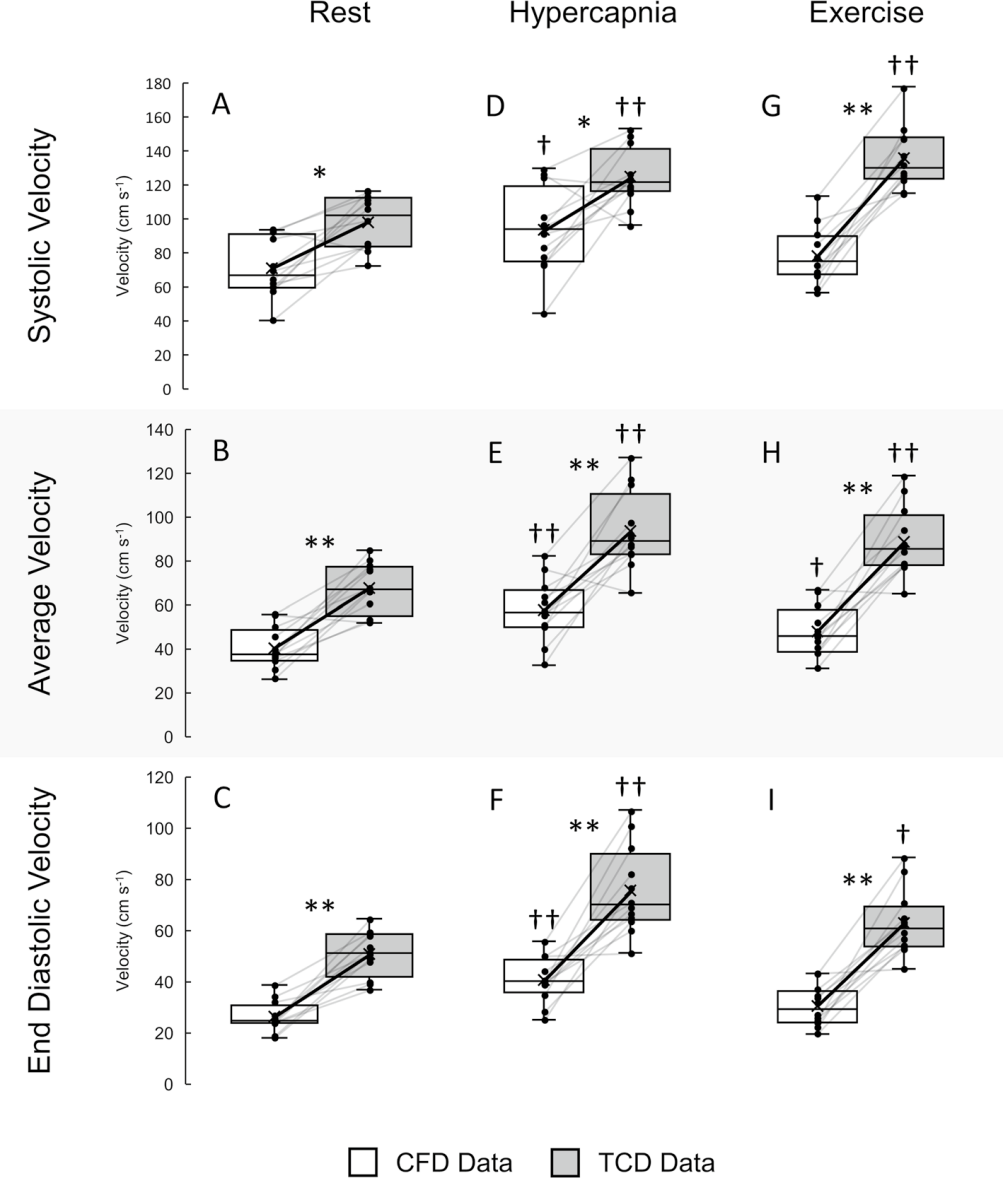
Distributions of systolic (**A**, **D**, **G**), average (**B**, **E**, **H**) and end-diastolic (**C**, **F**, **I**) velocity extracted from CFD (white) and TCD (grey) data in the right M1 segment. Individual differences between CFD and TCD data are presented as black markers with grey connecting lines. The solid black lines connecting the cross (X) in each box indicate the means of the respective distributions. These data were collected for each of the stimuli conditions of rest (**A**–**C**), hypercapnia (**D**–**F**) and exercise (**G**–**I**). Stars (*; inter-source between CFD and TCD data) and crosses (^†^; intra-source hypercapnia or exercise data compared to rest data) indicate the level of significance (* or ^†^*P* < 0.05; ** or ^††^*P* < 0.001) using paired *t*-tests

Distributions of CFD and TCD-derived velocity waveform metrics across the different exposure conditions are presented in Fig. 5. In response to hypercapnia and relative to rest, both CFD and TCD data exhibited significant increases (CFD: *P* range 0.001–0.003; TCD: all *P* < 0.001) across all velocity metrics relative to their respective resting measures. In response to exercise, all TCD velocity metrics increased significantly compared to rest (*P* range < 0.001–0.005), whereas only CFD average velocities (Fig. 5H) increased significantly compared to rest (40.4 ± 9.4 vs. 48.1 ± 11.3, *P* = 0.039; remainder *P* range 0.120–0.129). Across all conditions, measurements of maximal systolic, average and end-diastolic velocities were all significantly higher (*P* range < 0.001–0.004) in the TCD data compared to the CFD simulations. The distribution means of systolic velocities ranged from 33 to 73% higher in the TCD data compared to CFD across the exposure conditions. Distribution means were also higher for average velocities, with increases between 62 and 85% when comparing TCD to CFD data across all exposure conditions. The highest differences were present in end-diastolic velocities, with increases ranging from 85 to 106% between TCD and CFD data across all conditions. Statistical power analysis for inter-source (CFD vs TCD) and intra-source to rest (responses vs. rest) comparisons are given in Tables 1 and 2 of online resource 3.

**Fig. 6:**
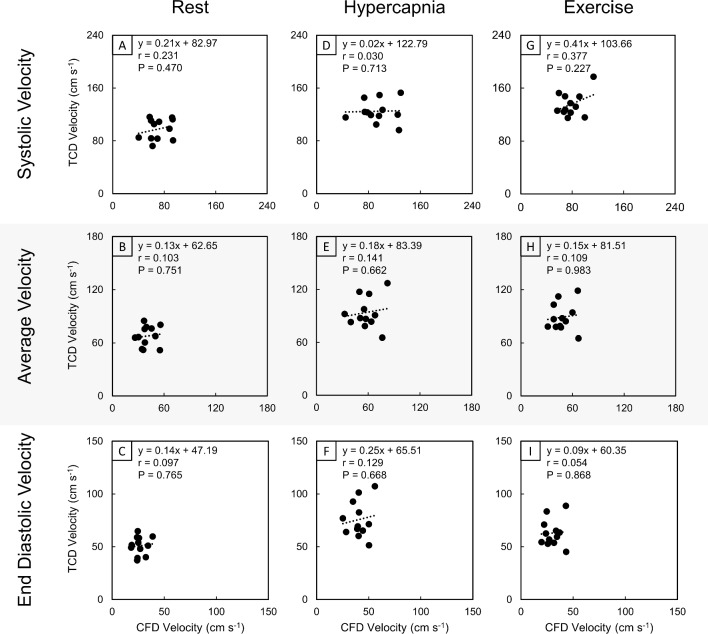
Correlation plots for absolute systolic (**A**, **D**, **G**), average (**B**, **E**, **H**) and end-diastolic (**C**, **F**, **I**) maximal velocity extracted from CFD and TCD data in the right M1 segment of the MCA. These data are collected from the rest (**A**–**C**), hypercapnia (**D**–**F**) and exercise (**G**–**I**) conditions. The linear regression equation (*y*), Pearson’s correlation coefficient (*r*) and *P*-value (*P*) are displayed for each correlation plot

Correlations of systolic, average and end-diastolic maximal velocities extracted from CFD and TCD waveforms, under the conditions of rest (Fig. 6A–C), hypercapnia (Fig. 6D–F) and exercise (Fig. 6G, H), were generally uncorrelated and exhibited weak positive relationships that were not significant (*r* range 0.030–0.377, *P* range 0.227–0.925). The corresponding Bland–Altman plots are given in online resource 4.

### Relative change in MCA velocity data distributions and correlations in response to physiological stimuli

**Fig. 7:**
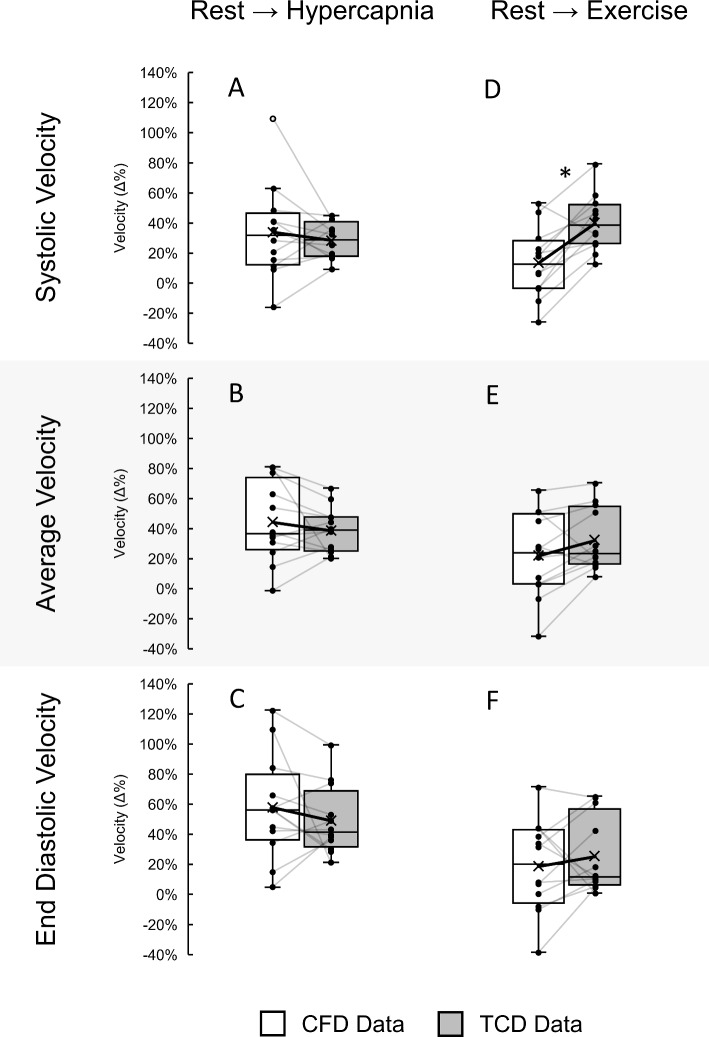
Distributions of the relative change (Δ%) in systolic, average and end-diastolic velocity extracted from CFD (white) and TCD (grey) data between responses from rest to hypercapnia (**A**–**C**) and to exercise (**D**–**F**) in the right M1 segment of the MCA. Individual differences between CFD and TCD relative change data are presented as black markers with grey connecting lines. The solid black line connecting the cross (X) in each box indicates the changing means of the distributions. A CFD data point of relative change in systolic velocity from rest to hypercapnia that is outside the higher quartile range limit is displayed as a hollow circle (o). Stars (*) indicate the level of significance (*P < 0.05; **P < 0.001; between CFD and TCD data) using paired *t*-tests

We investigated the distributions of the relative changes in velocity waveform metrics from rest to hypercapnia and rest to exercise between the CFD and TCD datasets (Fig. 7). We found that the distributions of the relative change in velocity metrics from rest to hypercapnia were similar and not significantly different between CFD and TCD (*P* range 0.364–0.482) data, with the means of relative velocity changes for CFD data higher than TCD data for systolic (Fig. 7A: 34 ± 30% vs. 28 ± 12%, *P* = 0.579), average (Fig. 7B: 44 ± 26% vs. 39 ± 15%, *P* = 0.532) and end-diastolic velocities (Fig. 7C: 58 ± 33% vs. 49 ± 22%, *P* = 0.477). One case exhibited a relative change in systolic velocity from CFD data in response to hypercapnia greater than the higher quartile range limit. While small differences between CFD and TCD distributions were observed for average (Fig. 7E: 22 ± 27% vs. 32 ± 20%, *P* = 0.323) and end-diastolic (Fig. 7F: 19 ± 29% vs. 26 ± 24%, *P* = 0.566) velocities in response to exercise, the changes in systolic velocities were significantly higher in the TCD data compared to CFD (Fig. 7D: 40 ± 18% vs. 14 ± 22%, *P* = 0.006). Statistical power analysis for inter-source (CFD vs TCD) relative change comparison is given in Table 3 of online resource 3.

**Fig. 8:**
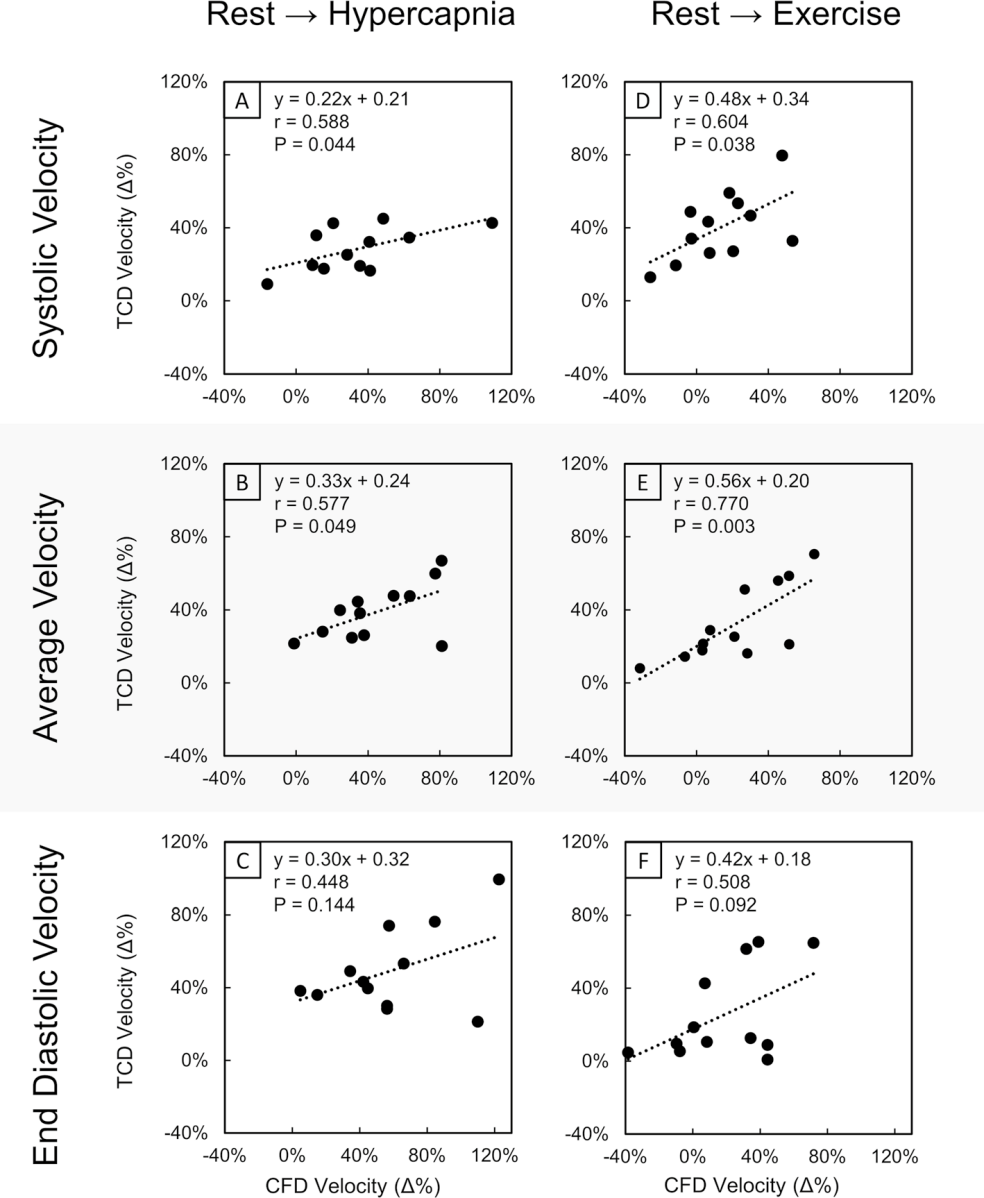
Correlation plots of the relative change (Δ%) in systolic (**A**, **D**), average (**B**, **E**) and end-diastolic (**C**, **F**) maximal velocity extracted from CFD and TCD data in the right M1 segment of the MCA for responses from rest to hypercapnia (**A**–**C**) and from rest to exercise (**D**–**F**). The linear regression equation (*y*), Pearsonʼs correlation coefficient (*r*) and *P*-value (*P*) are displayed for each correlation plot

In comparison with absolute data, correlations of CFD to TCD data for the relative change in the velocity waveform metrics of systolic, average and end-diastolic velocity from rest to hypercapnia and rest to exercise yielded moderate positive correlations (Fig. 8). Changes in systolic (Fig. 8A: *r* = 0.588, *P* = 0.044) and average (Fig. 8B: *r* = 0.577, *P* = 0.049) velocities from rest to hypercapnia were significantly correlated, while end-diastolic velocities (Fig. 8C: *r* = 0.448, *P* = 0.144) were not significantly correlated. Similarly, for the change from rest to exercise, relative changes in systolic (Fig. 8D: *r* = 0.604, *P* = 0.038) and average (Fig. 8E: *r* = 0.770, *P* = 0.003) velocities were found to be significantly correlated between CFD and TCD data, while end-diastolic (Fig. 8F: *r* = 0.508, *P* = 0.092) velocities were not significantly correlated. The corresponding Bland–Altman plots are given in online resource 5. Excluding relative change in systolic velocity bias from rest to exercise (− 27%), biases fell within ± 10% for all other velocity metrics and exposure conditions. A mild positive proportional bias was observed across most relative velocity change metrics.

## Discussion

Although CFD has previously been compared with TCD methods under static conditions (Groen et al. 2018; Panerai et al. 2016; Rivera et al. 2016; Shen et al. 2020), few studies have compared these approaches in response to physiological stimuli (Coverdale et al. 2014). This study aimed to investigate waveform velocities at rest and during hypercapnia and exercise. We compared measurements derived from TCD to velocities calculated via CFD simulations (derived from 3D MRA combined with ICA and VA duplex ultrasound). In contrast to our hypothesis, we observed that CFD and TCD-derived waveform velocity metrics were different and did not correlate at rest, but that changes in response to physiological stimuli were similar and significantly correlated.

### Differences between TCD and CFD data

The higher velocity data we observed using TCD compared to CFD at rest could potentially be explained by overestimation from TCD methods, underestimation from CFD simulations or a combination thereof. Although TCD velocity is a well-established method for measuring MCA BFv, higher TCD velocities have been observed previously due to phenomenon such as spectral broadening, which can exaggerate peak BFv by up to 35% when lower MHz probes are used (Eicke et al. 1995; Hoskins 1996). When comparing BFv between TCD and MRI-based methods, Seitz et al. (2001) found that TCD velocities exceeded MRA velocities by around 30%, and they also reported low correlations between these approaches. Chang et al. (2011) also reported 30% greater velocities via TCD, but found that phase-contrast MRA techniques correlated strongly with TCD. Conversely, a study by Meckel et al. (2013) compared 4D phase-contrast MRI and transcranial colour-coded duplex sonography and also observed that TCD-derived data were higher, with weak to mild correlations between these approaches. Leung et al. (2013) also reported higher peak velocities using TCD than phase-contrast MRA, but reported strong correlations when data were compared between approaches. Taken together, these studies indicate that, at rest and in response to some physiological stimuli, TCD approaches generate higher velocities compared to MRI-based methods, and the degree to which they correlate is variable. In our study, we also found higher TCD values, although our differences in comparison with CFD were larger than those previously reported for TCD versus MRI methods, suggesting that additional factors (discussed below) may explain the discrepancies we observed.

We also observed that TCD-derived velocities were uncorrelated with total duplex ultrasound flow measured in the ICAs and VAs (i.e. tCBF). This is despite both approaches being based on Doppler ultrasound. This suggests that cerebral autoregulation may affect MCA BFv in a manner distinct from any impact on extracranial ICA and VA blood flows. Hypercapnia is generally considered to induce MCA dilation (Coverdale et al. 2014; Coverdale et al. 2015; Verbree et al. 2014; Willie et al. 2012), and changes in MCA diameter could increase the variability of measured TCD velocities, compared to the corresponding ICA and VA duplex ultrasound-derived flows. It is important to note, however, that MCA diameter change due to ventilatory effects is debated in the literature (Ainslie and Hoiland 2014; Brothers and Zhang 2016; Hoiland and Ainslie 2016; Willie et al. 2012), with some studies reporting that MCA diameter does not change in response to hypercapnia (Serrador et al. 2000). Hence, hypercapnic responses may vary between individuals and study populations. Furthermore, while hypercapnia may dilate the ICA and VA, the mechanisms responsible may relate to increased shear stress secondary to downstream intra-cranial dilation, rather than direct effects of CO_2_ which are more apparent in the MCA. Distinct time courses of dilation may contribute to the lack of correlation in responses we observed between these arteries and techniques. Interestingly, in response to exercise, cerebral vessels can undergo vasoconstriction, associated with hyperventilatory effects (Ogoh and Ainslie 2009), with the magnitude of diameter change being smaller than that associated with hypercapnia (Verbree et al. 2017). This may explain our observation that, in contrast to our hypercapnic findings, changes in TCD-derived MCA time-averaged BFv in response to exercise were significantly correlated with tCBF based on ICA/VA duplex ultrasound (Fig. 4E: *r* = 0.588, *P* = 0.044).

Another potential factor affecting differences between CFD and TCD-derived data may relate to differences in posture. The MRA scans, and subsequent 3D geometries, used in the CFD simulations were collected with subjects supine. This contrasts with the semi-recumbent position during physiological testing (trunk angle ~ 60°). An increase in MCA diameter due to this difference in posture could contribute to lower calculated CFD MCA BFv, although data regarding postural impacts on MCA diameter remain limited and variable. Sato et al. (2012a) measured neck artery flows and MCA BFv using TCD in supine and 60° head-up tilt, finding that both MCA BFv and ICA volumetric blood flows were significantly higher supine. Garrett et al. (2017) also found that MCA BFv was significantly higher supine compared to upright. Serrador et al. (2000) used a combination of TCD and MRI to measure MCA BFv and diameter in supine participants who underwent simulated orthostatic stress via lower body negative pressure, finding that although MCA BFv was significantly higher supine compared to during lower body pressure, no significant changes in MCA diameter were observed. Despite documented changes in velocity in the MCA between postures, the limited evidence reviewed above suggests that MCA diameter may not change in distinct postures. Therefore, differences in calculated CFD BFv may be more attributable to the incoming blood flow, rather than postural variations in MCA lumen diameter.

The differences we observed between TCD and CFD calculated MCA BFv data cannot be ascribed to differences between the CFD data and duplex ICA and VA-derived blood flow, as the latter were highly correlated. This is not surprising, given that duplex ICA and VA-derived blood flows were inputs for the CFD simulations from which CFD MCA BFv data were derived. Indeed, ICA and VA flows derived from duplex ultrasound have been found to correlate with MRI-based estimates (Khan et al. 2017; Oktar et al. 2006). The average ICA and VA ultrasound flows collected in our study were similar to previous studies at rest and during exposure to stimuli (Oktar et al. 2006; Sato et al. 2012b; Skytioti et al. 2016; Steventon et al. 2018; Tallon et al. 2022; Willie et al. 2012).

In summary, we found that MCA BFv derived from TCD was higher than that calculated from CFD, potentially due to TCD-related phenomena such as spectral broadening. In addition, we observed that TCD BFv metrics were not correlated with extracranial duplex ultrasound-derived flows in the ICAs and VAs. In contrast, CFD calculated BFv was significantly correlated with ICA and VA flows at rest and in response to stimuli. Nonetheless, future studies interested in using CFD to aid in calculating velocity, shear stress, oscillatory shear and other important cerebrovascular haemodynamics in response to physiological stimuli may require further model refinement.

### Relative change responses between TCD and CFD data

Despite the differences between TCD and CFD measured in absolute terms that we describe above, an arguably more important question is whether changes in velocity responses to physiological stimuli derived using each technique are correlated. We observed that relative changes from baseline data in response to hypercapnia and exercise were similar and highly correlated in our study when TCD and CFD approaches were compared. Comparison of relative changes in velocity may minimise the impact of systematic sources of variability in either method, since relative changes could serve to reduce consistent within-subject error through normalisation.

In concert with this, it is plausible that inherent physiological variability between individuals may be reduced when a standardised stimulus (hypercapnia, exercise) is applied. Hypercapnia induced similar changes in velocity metrics between TCD and CFD sources. In response to exercise, average velocity was also similar, but systolic velocity was higher by TCD assessment (in keeping with findings above). These data suggest that hypercapnia may induce a more consistent response among individuals compared to exercise, which in comparison is a compound and complex stimulus. Nonetheless, relative changes in systolic and average MCA BFv derived from TCD and CFD approaches, in response to both hypercapnia and exercise, were significantly correlated. Our findings therefore indicate that TCD or CFD methods result in similar changes in physiological responses in MCA BFv in humans. CFD simulations therefore provide relative change data, which is consistent with that derived from TCD, with the added benefit of being able to derive further haemodynamic metrics such as the change in time-averaged wall shear stress or oscillatory shear in response to stimuli such as hypercapnia and exercise.

### Limitations

Due to fundamental limitations in MRI scanning, the axial slice thickness direction is likely aligned with the cross section of the MCA in most cases. This may lead to reduced resolution in the MRA scans about the MCA cross section, which may then influence the flow in the CFD simulations and hence the CFD derived MCA velocities. Given the nature of the imaging modality used and orientation required for collecting images, this variation in slice thickness is difficult to account for, but could be rectified by manually adjusting the MCA cross-sectional region of the 3D models using surface meshing tools to reflect a diameter from an axial plane selected closest to the centreline of the MCA. Additionally, and as discussed above, MRA scanning was performed in resting supine participants. Ideally, provided availability of specialised equipment, MRA scans should be captured during or in response to stimuli, allowing any individual changes in vessel diameters to be embedded into each of the CFD simulations. Similarly, true validation of CFD methods was unable to be performed in this study. Independently captured time-varying image datasets using 4D flow MRI methods may provide additional validation for future cerebrovascular CFD simulations.

While we used previously established methods (Kurmanavichius et al. 1989; Thomas et al. 2020) for ultrasound waveform averaging, additional averaging of cardiac cycles may also serve to reduce the variability in results. Similarly, the flow in the ICAs and VAs was calculated using previously established methods assuming Poiseuille flow (Tallon et al. 2022; Thomas et al. 2020). Although flow calculation using this method has been found to be relatively consistent with Womersley-derived flow in larger arteries such as the common carotid (Mynard and Steinman 2013), it may nonetheless have contributed to flow variation. Future CFD simulations should consider calculating Womersley-derived flow from duplex ultrasound data.

In the CFD simulations, outlet boundary conditions were distributed using resting regional flow measurements derived from the literature (i.e. WALNUT) and diameter-based flow splitting exponents which were constant across all outlets. In the absence of regional brain blood flow data, particularly in response to stimuli, an exponent value appropriate for cerebrovascular vessels was used (Thomas et al. 2020). However, research has suggested that this exponent may vary for each individual outlet (Chnafa et al. 2017, 2018). A localised outlet splitting method as described by Chnafa et al. (2018), provided access to measured flow data in the brain, may be more appropriate in future CFD-based cerebrovascular research.

In addition, the CFD simulations employed rigid wall modelling. Alternatively, implementation of fluid structure interaction (FSI) modelling would allow the vessel wall to become compliant and to deform and absorb energy throughout the cardiac cycle. However, we did not implement FSI modelling as the resolution of the MRA data collected was unable to resolve arterial wall thickness and subject-specific material properties were not known—factors which could have introduced further sources of variability to the simulations. Interestingly, due to absorption of fluid energy, FSI modelling would likely reduce the velocities calculated from CFD simulation—and thus likely exacerbate the discrepancies we observed between TCD and CFD velocities in this study.

Finally, with only 12 participants, the number of cases investigated in this study is relatively small and was limited to young, healthy individuals with no pre-existing cardiovascular diseases. Consequently, future studies should consider larger sample sizes to further reduce the variability associated with either technique. However, despite the low number of cases, we nonetheless observed statistically significant results which may have important implications for future physiological research.

## Conclusion

In this study, we aimed to compare data in the cerebral vasculature under resting conditions, and in response to physiological stimuli (hypercapnia, exercise), using TCD ultrasound and independently constructed flow-conserving CFD simulations. Although we found differences between absolute velocity data obtained between CFD and TCD, measurements of relative change in velocity in response to stimuli showed good agreement, particularly for relative changes in time-averaged velocity. Therefore, in addition to absolute measurements, investigation of relative changes in velocity in response to physiological stimuli may be an important tool for future research using TCD ultrasound or for haemodynamic analysis using CFD cerebral vasculature simulations.

### Supplementary Information

Below is the link to the electronic supplementary material.

Supplementary file1 (PDF 13637 KB)

Supplementary file2 (TIF 5557 KB)

Supplementary file3 (PDF 212 KB)

Supplementary file4 (TIF 1585 KB)

Supplementary file5 (TIF 1381 KB)
