# Supplementary material for: Comparison of computational fluid dynamics with transcranial Doppler ultrasound in response to physiological stimuli
Source: Biomech Model Mechanobiol. 2023 Oct 8;23(1):255–69. doi: 10.1007/s10237-023-01772-9 (PMC10902019; doi:10.1007/s10237-023-01772-9)
Supplement: Supplementary file 1 — Supplementary file1 (PDF 13637 KB) [file 10237_2023_1772_MOESM1_ESM.pdf]

Comparison of Computational Fluid Dynamics with Transcranial Doppler Ultrasound in Response to Physiological Stimuli – Online Resource 1

Harrison T. Caddy[1,2], Hannah J. Thomas[2], Lachlan J. Kelsey[1,3], Kurt J. Smith[2,4], Barry J. Doyle[1,3*] and Daniel J. Green[2*]

[1]*Vascular Engineering Laboratory*, Harry Perkins Institute of Medical Research, Queen Elizabeth II Medical Centre, Nedlands, Australia and the UWA Centre for Medical Research, The University of Western Australia, Perth, Australia
[2]*School of Human Sciences (Exercise and Sport Sciences)*, The University of Western Australia, Perth, Australia
[3]*School of Engineering*, The University of Western Australia, Perth, Australia
[4]*Cerebrovascular Health, Exercise, and Environmental Research Sciences Laboratory, University of Victoria, Victoria, Canada*

* Joint senior authors

Acknowledgements

We acknowledge the resources provided by the Pawsey Supercomputing Centre with funding from the Australian Government and the Government of Western Australia. H.T.C is supported by a Forrest Research Foundation Scholarship and Australian Government Research Training Program Scholarship at The University of Western Australia. D.J.G. is supported by a National Health and Medical Research Council Principal Research Fellowship (APP1080914).

AUTHOR FOR CORRESPONDENCE:

Associate Professor Barry J Doyle

barry.doyle@uwa.edu.au

The University of Western Australia (M519),

35 Stirling Highway, 6009 Perth, Australia

CFD Simulation Boundary Condition Specification

An overview of the boundary condition specifications used and volume mesh can be seen in **Fig. 1**.

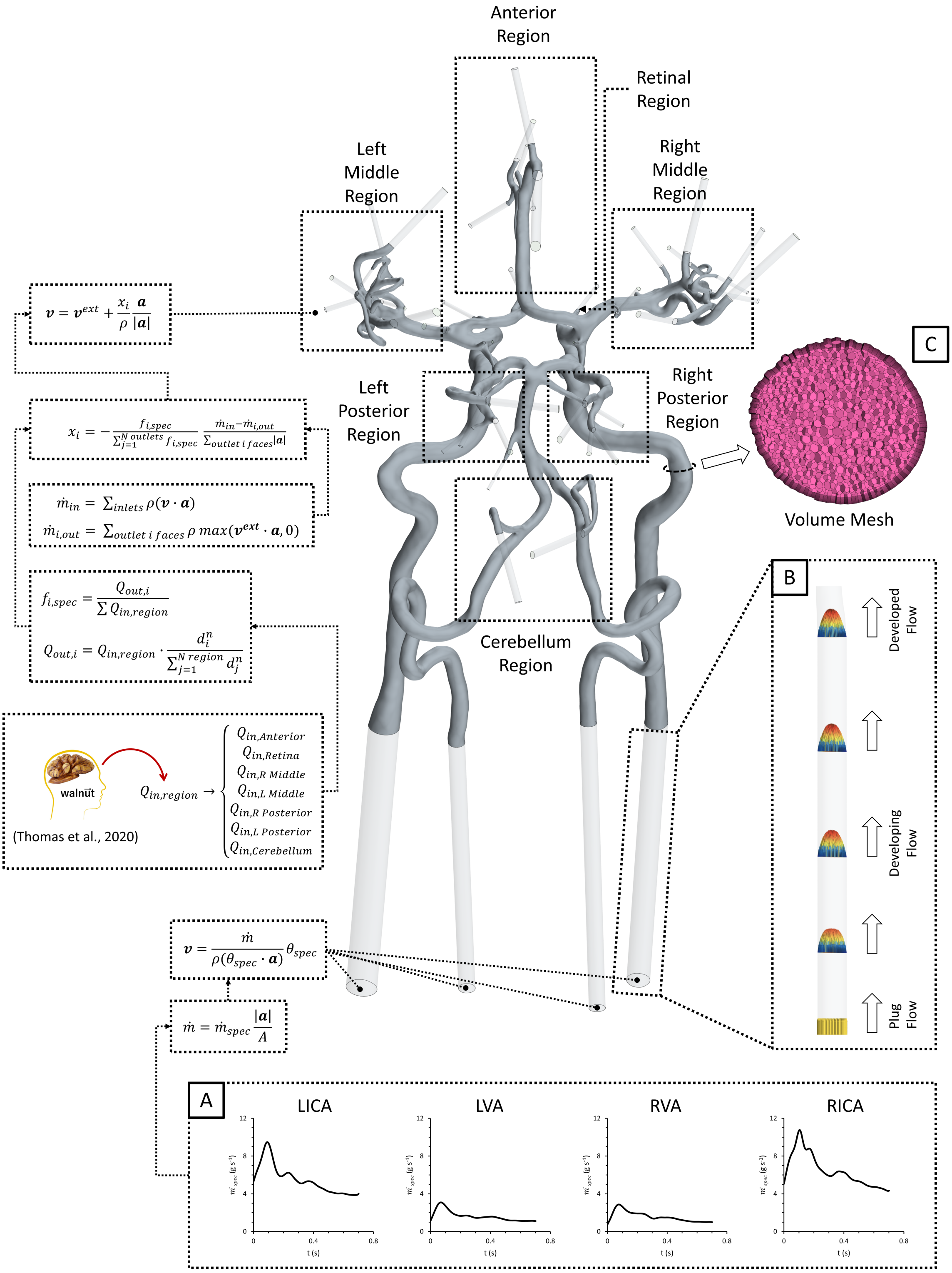

Fig. 1 Overview of boundary condition specifications at the inlets and outlets, along with examples of measured inlet mass flow waveforms (A) using duplex ultrasound for the left (LICA) and right (RICA)

internal and left (LVA) and right (RVA) vertebral arteries, flow development within the extrusions applied at the inlets to the domain (B) and an example cross-section of the volume mesh of the domain (C). Additionally, the general 7 cerebrovascular regions (Anterior, Retina, Right and Left Middle, Right and Left Posterior and Cerebellum) of which outlets were assumed to flow to are also shown

As depicted in **Fig. 1**, the flow waveforms for each incoming left and right internal (ICA) and vertebral (VA) arteries measured from duplex ultrasound (for each condition of rest, hypercapnia and exercise respectively) were converted to mass flow waveforms (**Fig. 1A**) assuming a density (ρ) of 1050 kg m^{-3} (Levitt et al. 2017). These waveforms were then specified as the incoming mass flow value ($\dot{m}_{spec}$) for each inlet, which was applied using the mass flow inlet condition within STAR-CCM+. For each inlet, a face averaged mass flow vector ($\dot{m}$) is calculated as per equation 1.

$$\dot{m} = \dot{m}_{spec} \frac{|\boldsymbol{a}|}{A} \quad (1)$$

Where $\boldsymbol{a}$ is the outwards pointing face area vector and A is the total area of the boundary.

STAR-CCM+ then implements the mass flow boundary condition using a face velocity vector ($\boldsymbol{v}$) at the boundary as per equation 2, resulting in "plug" flow profile demonstrated in (**Fig. 1B**).

$$\boldsymbol{v} = \frac{\dot{m}}{\rho(\theta_{spec} \cdot \boldsymbol{a})} \theta_{spec} \quad (2)$$

Where θ_{spec} is the inflow direction. As depicted in **Fig. 1B**, the extrusions of 11 times the diameter (Bluestein et al. 1997) allow for the development of flow prior to entering the fluid domain – transitioning from a "plug" flow condition to a more parabolic shape.

Fluid is then transported throughout the domain, where an example of the domain volume mesh can be observed in **Fig. 1C** – consisting of a combination of polyhedral elements in the core of the mesh and prism layer elements at the wall boundary.

At the outlets the WALNUT code was first used, of which details and the code can be found in previous research by Thomas *et al.* (2020), to estimate the flow to the 7 different outlet regions

($Q_{in,\ region}$) within the brain (Regions: Anterior, Retina, Right and Left Middle, Right and Left Posterior and Cerebellum) based on each participant's time-average flows measured at the left and right ICAs and VAs for each respective condition (rest, hypercapnia and exercise). For a given outlet i, boundary specific split ratios of total incoming flow ($f_{i,spec}$) were calculated as per equation 3, where flow to an outlet within a region was assumed to be first split by the flow to the parent region ($Q_{in,\ region}$) and then within that region using the Murry's Law formulation with an exponent (n) of 2.33 (Equation 4).

$$f_{i,spec} = \frac{Q_{out,i}}{\sum Q_{in,\ region}} \tag{3}$$

$$Q_{out,i} = Q_{in,\ region} \cdot \frac{d_i^n}{\sum_{j=1}^{N\ region} d_j^n} \tag{4}$$

Where d is the diameter of an outlet.

The flow split ratio specification was then prescribed for each outlet within STAR-CCM+, which calculates a boundary mass-flux correction factor for each outlet (Equation 7) using mass flow values calculated on outlet (Equation 5) and inlet (Equation 6) boundaries, and the previously calculated boundary specific split ratio (Equation 3).

$$\dot{m}_{i,out} = \sum_{outlet\ i\ faces} \rho\ max(\boldsymbol{v^{ext}} \cdot \boldsymbol{a}, 0) \tag{5}$$

$$\dot{m}_{in} = \sum_{inlets} \rho(\boldsymbol{v} \cdot \boldsymbol{a}) \tag{6}$$

$$x_i = -\frac{f_{i,spec}}{\sum_{j=1}^{N\ outlets} f_{i,spec}} \frac{\dot{m}_{in} - \dot{m}_{i,out}}{\sum_{outlet\ i\ faces} |\boldsymbol{a}|} \tag{7}$$

Where $\boldsymbol{v^{ext}}$ is the velocity value extrapolated from the cell adjacent to the boundary.

The flow split ratio specification then prescribes the face velocity vector at a given outlet i using equation 8.

$$\boldsymbol{v} = \boldsymbol{v^{ext}} + \frac{x_i}{\rho} \frac{\boldsymbol{a}}{|\boldsymbol{a}|} \tag{8}$$

References

Bluestein D, Niu L, Schoephoerster RT, Dewanjee MK (1997) Fluid mechanics of arterial stenosis: Relationship to the development of mural thrombus. Annals of Biomedical Engineering 25:344. https://doi.org/10.1007/BF02648048

Levitt MR et al. (2017) Computational fluid dynamics of cerebral aneurysm coiling using high-resolution and high-energy synchrotron X-ray microtomography: comparison with the homogeneous porous medium approach. Journal of neurointerventional surgery 9:0-0. https://doi.org/10.1136/neurintsurg-2016-012479

Thomas HJ et al. (2020) Assessment of cerebrovascular responses to physiological stimuli in identical twins using multimodal imaging and computational fluid dynamics. Journal of Applied Physiology 129:1024-1032. https://doi.org/10.1152/japplphysiol.00348.2020
