# Supplementary material for: Comparison of computational fluid dynamics with transcranial Doppler ultrasound in response to physiological stimuli
Source: Biomech Model Mechanobiol. 2023 Oct 8;23(1):255–69. doi: 10.1007/s10237-023-01772-9 (PMC10902019; doi:10.1007/s10237-023-01772-9)
Supplement: Supplementary file 3 — Supplementary file3 (PDF 212 KB) [file 10237_2023_1772_MOESM3_ESM.pdf]

Comparison of Computational Fluid Dynamics with Transcranial Doppler Ultrasound in Response to Physiological Stimuli – Online Resource 3

Harrison T. Caddy[1,2], Hannah J. Thomas[2], Lachlan J. Kelsey[1,3], Kurt J. Smith[2,4], Barry J. Doyle[1,3*] and Daniel J. Green[2*]

[1]*Vascular Engineering Laboratory*, Harry Perkins Institute of Medical Research, Queen Elizabeth II Medical Centre, Nedlands, Australia and the UWA Centre for Medical Research, The University of Western Australia, Perth, Australia
[2]*School of Human Sciences (Exercise and Sport Sciences)*, The University of Western Australia, Perth, Australia
[3]*School of Engineering*, The University of Western Australia, Perth, Australia
[4]*Cerebrovascular Health, Exercise, and Environmental Research Sciences Laboratory, University of Victoria, Victoria, Canada*

* Joint senior authors

Acknowledgements

We acknowledge the resources provided by the Pawsey Supercomputing Centre with funding from the Australian Government and the Government of Western Australia. H.T.C is supported by a Forrest Research Foundation Scholarship and Australian Government Research Training Program Scholarship at The University of Western Australia. D.J.G. is supported by a National Health and Medical Research Council Principal Research Fellowship (APP1080914).

AUTHOR FOR CORRESPONDENCE:

Associate Professor Barry J Doyle

barry.doyle@uwa.edu.au

The University of Western Australia (M519),

35 Stirling Highway, 6009 Perth, Australia

Table 1 Paired t-test two tailed power analysis between distributions of CFD and TCD maximal velocity waveform characteristics (systolic, average and end diastolic).

	Rest	Hypercapnia	Exercise
**Systolic Velocity**			
d_{av}	1.63	1.45	3.30
Power (CFD vs TCD)	1.00	0.99	1.00
**Average Velocity**			
d_{av}	2.61	2.25	2.97
Power (CFD vs TCD)	1.00	1.00	1.00
**End Diastolic Velocity**			
d_{av}	3.25	2.57	3.06
Power (CFD vs TCD)	1.00	1.00	1.00

Effect size calculated as Cohen's d using average variance (d_{av}) with a paired sample count of n=12.

Table 2 Paired t-test two tailed power analysis of stimuli (hypercapnia or exercise) response distributions of CFD or TCD maximal velocity waveform characteristics (systolic, average and end diastolic) relative to the corresponding rest condition.

	Hypercapnia vs Rest	Exercise vs Rest
**Systolic Velocity**		
CFD Data Power d_{av}	1.05	0.43
CFD Data Power	0.91	0.28
TCD Data Power d_{av}	3.13	3.66
TCD Data Power	1.00	1.00
**Average Velocity**		
CFD Data Power d_{av}	1.45	0.74
CFD Data Power	1.00	0.65
TCD Data Power d_{av}	3.75	3.73
TCD Data Power	1.00	1.00
**End Diastolic Velocity**		
CFD Data Power d_{av}	1.90	0.59
CFD Data Power	1.00	0.46
TCD Data Power d_{av}	3.85	3.67
TCD Data Power	1.00	1.00

Effect size calculated as Cohen's d using average variance (d_{av}) with a paired sample count of n=12.

Table 3 Paired t-test two tailed power analysis between relative change distributions of CFD and TCD maximal velocity waveform characteristics (systolic, average and end diastolic) from rest to hypercapnia and rest to exercise.

	Rest → Hypercapnia	Rest → Exercise
**Systolic Velocity**		
d_{av}	0.23	1.26
Power (CFD vs TCD)	0.11	0.98
**Average Velocity**		
d_{av}	0.26	0.41
Power (CFD vs TCD)	0.13	0.26
**End Diastolic Velocity**		
d_{av}	0.30	0.24
Power (CFD vs TCD)	0.16	0.12

Effect size calculated as Cohen's d using average variance (d_{av}) with a paired sample count of n=12.
