# Supplementary figures and images for: Comparison of computational fluid dynamics with transcranial Doppler ultrasound in response to physiological stimuli
Source: Biomech Model Mechanobiol. 2023 Oct 8;23(1):255–69. doi: 10.1007/s10237-023-01772-9 (PMC10902019; doi:10.1007/s10237-023-01772-9)

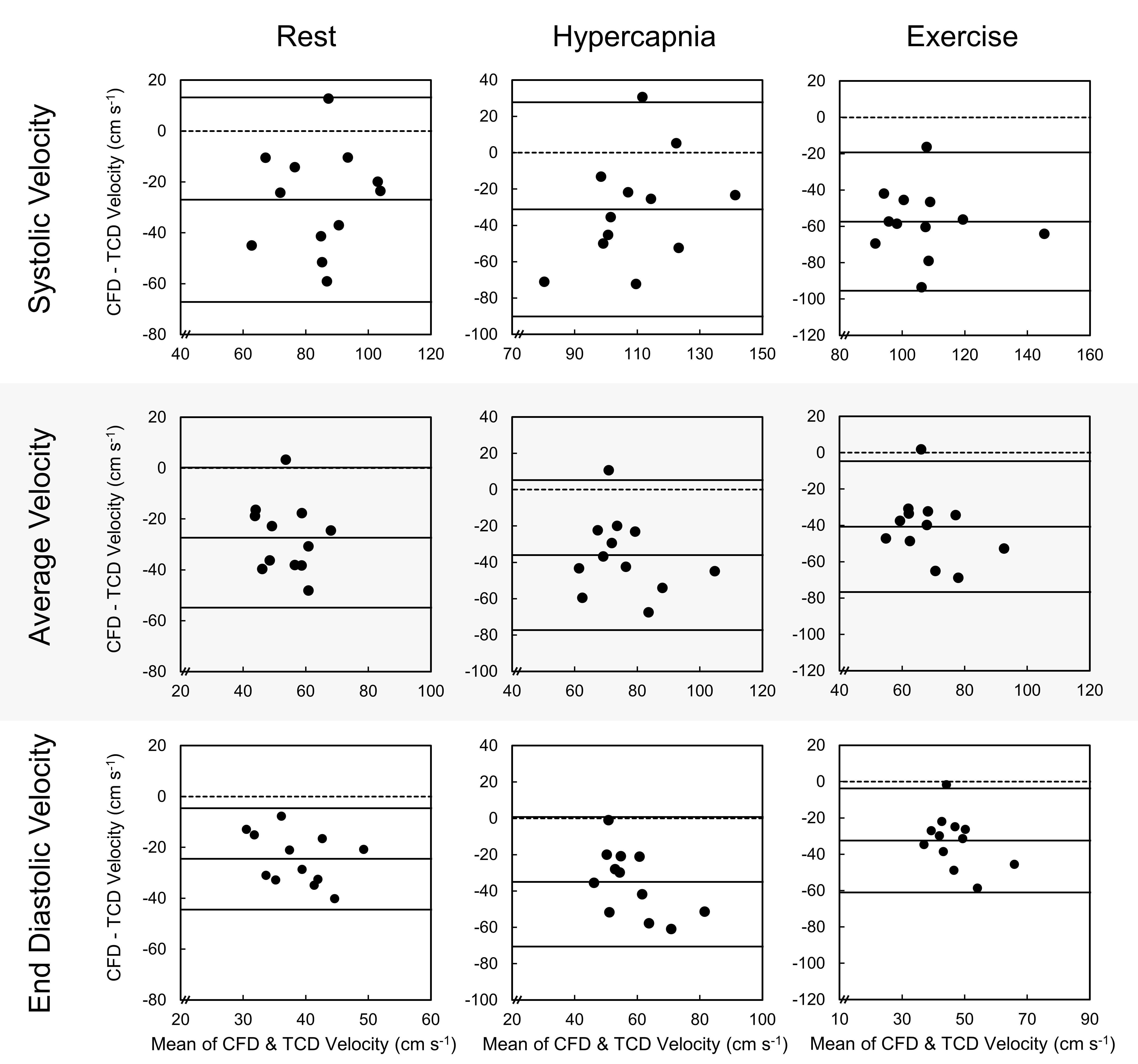

Supplement: Supplementary file 4 — Supplementary file4 (TIF 1585 KB) [file 10237_2023_1772_MOESM4_ESM.tif]

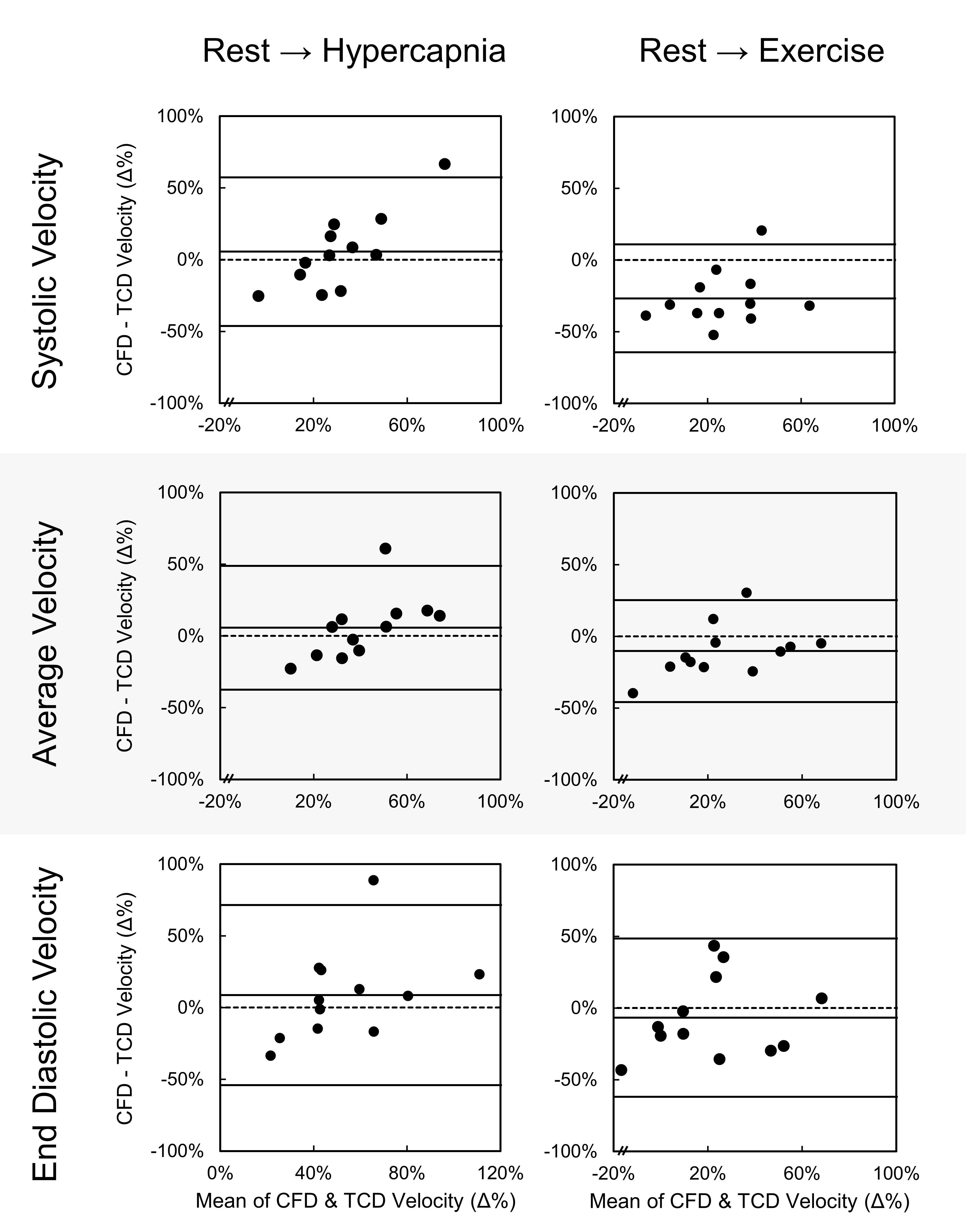

Supplement: Supplementary file 5 — Supplementary file5 (TIF 1381 KB) [file 10237_2023_1772_MOESM5_ESM.tif]
